# New Insights into Regulation of Proteome and Polysaccharide in Cell Wall of *Elsholtzia splendens* in Response to Copper Stress

**DOI:** 10.1371/journal.pone.0109573

**Published:** 2014-10-23

**Authors:** Tingting Liu, Chaofeng Shen, Yi Wang, Canke Huang, Jiyan Shi

**Affiliations:** Institute of Environmental Science and Technology, College of Environmental and Resource Sciences, Zhejiang University Hangzhou, P.R. China; Institute for Sustainable Plant Protection, C.N.R., Italy

## Abstract

**Background and Aims:**

Copper (Cu) is an essential micronutrient for plants. However, excess amounts of Cu are toxic and result in a wide range of harmful effects on the physiological and biochemical processes of plants. Cell wall has a crucial role in plant defense response to toxic metals. To date, the process of cell wall response to Cu and the detoxification mechanism have not been well documented at the proteomic level.

**Methods:**

An recently developed 6-plex Tandem Mass Tag was used for relative and absolute quantitation methods to achieve a comprehensive understanding of Cu tolerance/detoxification molecular mechanisms in the cell wall. LC–MS/MS approach was performed to analyze the Cu-responsive cell wall proteins and polysaccharides.

**Key Results:**

The majority of the 22 up-regulated proteins were involved in the antioxidant defense pathway, cell wall polysaccharide remodeling, and cell metabolism process. Changes in polysaccharide amount, composition, and distribution could offer more binding sites for Cu ions. The 33 down-regulated proteins were involved in the signal pathway, energy, and protein synthesis.

**Conclusions:**

Based on the abundant changes in proteins and polysaccharides, and their putative functions, a possible protein interaction network can provide new insights into Cu stress response in root cell wall. Cu can facilitate further functional research on target proteins associated with metal response in the cell wall.

## Introduction

Copper (Cu) is an essential micronutrient for plants because it is a structural and catalytic component of several proteins or enzymes involved in various physiological metabolisms [1]. However, excess Cu in plants is harmful; it induces phytotoxicity [2] and disturbs the physiological metabolisms, including carbohydrate metabolism, protein metabolism, mineral nutrition, cell respiration, and photosynthesis [3]. Given its redox properties, Cu^2+^ can catalyze the generation of reactive oxygen species (ROS) and Fenton reactions that damage cellular components or interfere with cellular transport processes [4]. Plants cannot avoid the stress conditions from Cu-contaminated areas. Thus, several resistance mechanisms for Cu tolerance have been developed [5], including exclusion, compartmentalization, chelation, and binding to organic ligands, which enable maintenance of Cu homeostasis for plant survival even in a highly contaminated environment [6].

Plant cell wall is the primary site for signal perception and defense response, which is significant in responding to environmental stresses [7]. The cell walls of plants that grow on heavy metal-contaminated areas provide physical barriers against toxic heavy metals and actively participate in plant defense response. The cell wall serves as a repository for high contents of metals; it senses stress signals and transmits them to the cell interior, thereby affecting the cell fate decision [8]. The main chemical components of a plant cell wall include polysaccharides and proteins. Polysaccharides represent up to 90% of the plant cell wall and constitute three different kinds of polymers, namely, cellulose, hemicelluloses, and pectins. Cell wall proteins (CWPs) of plants comprise less than 10% of the cell wall dry weight but have major roles in cell wall structure, metabolism, cell enlargement, signal transduction, defense responses, and many other physiological events [9].

Certain plants can accumulate high content of metal ions in their cell wall through various compounds. Cell wall polysaccharides possess crucial roles in heavy metal binding and accumulation. Binding of metal ions depends on the number of functional groups in the cell wall polysaccharides. Previous studies confirmed that the essential capacity of the cell wall for binding metal ions depends mainly on the abundance of polysaccharides in the carboxyl groups [10]. Current information about the regulation of cell wall polysaccharides under metal stress remains limited.

Abiotic stress induces expression of stress-responsive proteins, which lead to cellular adaptation in plant growth under stress conditions. Therefore, studies have been carried out to identify various CWPs involved in stress response, particularly those of regulatory or targeting function proteins [11]–[17]. Most reports have focused on leaf cell wall proteomes under abiotic stress, such as salt in tobacco [11]; wounding in *Medicago* [12]; dehydration in chickpea, rice, and maize [9], [14], [15]; zinc toxicity in *Hordeum* [18]; nickel toxicity in barley [19]; manganese toxicity in cowpea [20], [21]; boron deficiency in *Lupinus albus* [22]; and flooding in soybean [23]. Few studies have focused on the root cell wall proteomes under abiotic stress [24]–[26]. All of these studies broadened our understanding of the complicated regulation of apoplast proteins. An in-depth proteome study of the cell wall can provide extensive information on the intrinsic mechanism of stress response attributed to a possible relationship between protein abundance and plant stress tolerance [27].

*Elsholtzia splendens* is a plant that can survive in a highly Cu-polluted area. This species can accumulate more than 1000 mg/kg of Cu in the body [28], and the major proportion (70%) of Cu ions is stored in the root cell wall [29]. Nevertheless, little is known about the defense and detoxification mechanisms in the root cell wall of *E. splendens*. A recently developed 6-plex Tandem Mass Tag (TMT) was used for relative and absolute quantitation methods to gain a comprehensive understanding of Cu tolerance/detoxification molecular mechanisms. LC–MS/MS approach was also conducted to analyze Cu-responsive CWPs and polysaccharides in this study. This proteomic study of the apoplast is the first in response to Cu. The results will significantly expand knowledge on the apoplast complexicity in metal-tolerant plants and develop a more comprehensive understanding of the involved cellular reactions in cell defense mechanisms.

## Results

### Isolation and purification of CWPs

**Figure 1:**
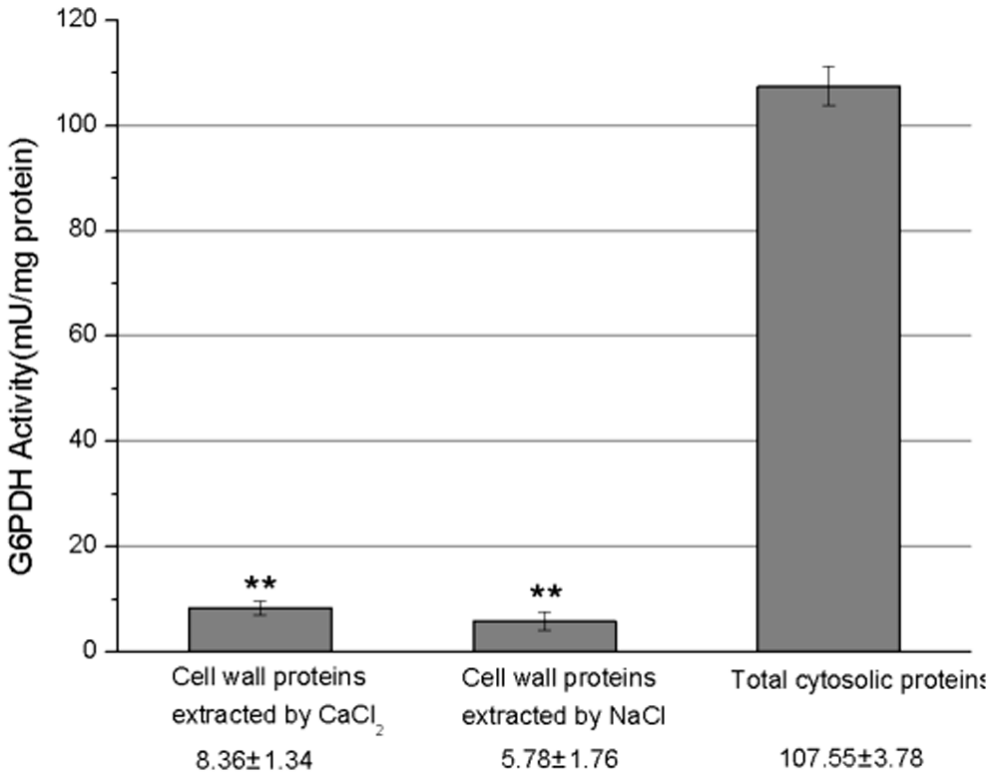
Evaluation of purity by the G6PDH activity in the cell wall proteins isolated from the roots of *Elsholtzia splendens*. The activity of G6PDH was assayed in CaCl_2_-extracted cell wall proteins, NaCl-extracted cell wall proteins and total soluble proteins. One unit of G6PDH activity is defined as 1 µmol of NADPH turnover per min/mg protein. Results are presented as mean ±SE of G6PDH activity from three biological replicates. The asterisks indicate significant differences in the G6PDH activity of CaCl_2_-extracted cell wall proteins, NaCl-extracted cell wall proteins compared with that of total soluble cytosolic proteins (**p<0.01).

Root CWPs from *E. splendens* were isolated with sucrose gradients and washed according to the method of Feiz et al. [30]. The activity of G’DH, a typical marker enzyme used to detect contamination of cytoplasmic proteins, was measured to validate the purification method [31]. The G6PDH activity in CaCl_2_-extracted CWPs, NaCl-extracted CWPs, and cytosolic protein fractions were 8.36, 5.78, and 107.55 mU/mg protein, respectively (Figure 1). The G6PDH activity in CaCl_2_- or NaCl-extracted CWPs was 10 times lower than that in cytosolic proteins (*p*<0.01). These results indicate that the contamination of intracellular proteins was minimal.

The identified proteins were classified into different gene ontology (GO) terms according to their cellular compartment(s), function(s), and biological processes (Table S1). Some of these identified proteins showed a compartment label (apoplast, cell wall, extracellular region, and plant-type cell wall) that is coherent with secreted proteins. The same proteins can also be attributed to various compartments in the GO classification. Three different computer programs (TargetP, Predotar, and WoLF PSORT) were used to predict the protein subcellular localization (Table S1). A protein was secreted when at least two out of the three algorithms predicted extracellular localization. Based on the results, 15 proteins were predicted to be secreted proteins in the fractions (Table S1).

### Cu-induced changes in root CWP contents

**Figure 2:**
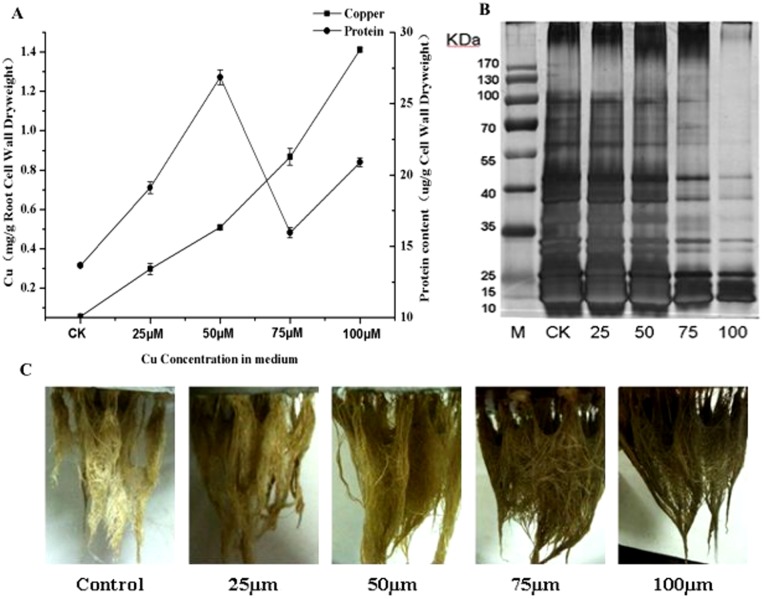
Effect of different copper concentration on the cell wall protein and copper ions in *Elsholtzia splendens*'s root cell. A: The content of copper and protein every root cell wall dry weight. B: SDS-PAGE of root cell wall protein under different copper stress. C: Root growth patterns of control and copper-stressed plants during different copper concentration. Data presented are mean ±SE (n = 20),*Significant mean differences from control at *p* = 0.05 in multiple comparison by LSD test.

Plants were grown on a medium supplemented with different CuSO_4_ concentrations to determine changes in CWP abundance under Cu stress (Figure 2A). Cu toxicity led to a strong increase in CWP contents at low Cu concentrations. The CWP contents increased 1.39-fold at 25 µM Cu and 1.96-fold at 50 µM Cu. The highest content of the CWPs was observed at 50 µM Cu concentrations. The plant root began to decompose at 100 µM Cu (Figure 2C). The increase of Cu contents in the cell wall could proportionally increase with the Cu supply in the medium (slope  = 0.3, *R* ^2^ = 0.98) at Cu concentrations below 75 µM. The Cu content in the root cell wall continued to increase significantly (slope  = 0.45, *R* ^2^ = 0.99) above 75 µM Cu. The highest value of Cu content in the root cell wall reached 1.41 mg/g with the root cell wall dry weight at 100 µM Cu. These findings indicate an increase in Cu binding to cell walls with Cu supply in the medium (Figure 2A). Polypeptide separation by SDS-PAGE (Figure 2B) revealed about 30 polypeptide bands. Band intensities increased with the increase in Cu concentration below 50 µM (Figure S1). The staining intensity was the lowest among all the cell wall polypeptides at 100 µM Cu concentration.

### Cu-induced remodeling in root cell wall polysaccharide

**Figure 3:**
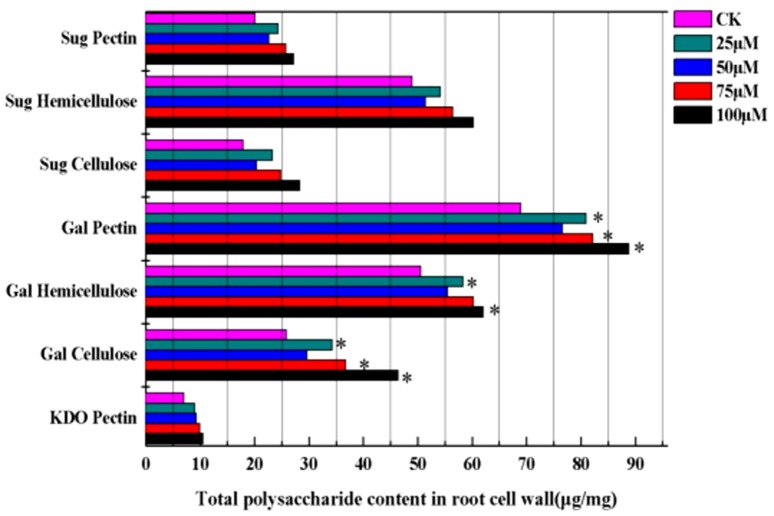
Effect of different copper concentrations on the contents of root cell wall polysacchride. Sug: sugar, Gal: galacturonic acids, KDO: 2-Keto-3-deoxyoctonic acid. Data presented are mean ±SE (n = 3). *Significant mean difference from control at *p* = 0.05 in multiple comparison by LSD test.

Evidence generally suggests that cell wall polysaccharides are crucial sites for Cu retention in plants. Therefore, changes in the contents of root cell wall polysaccharide of *E. splendens* were measured. Treatments with 25, 50, 75, and 100 µM Cu significantly increased the expression of CWPs and polysaccharides compared with controls (Figure 3). Cu supply enhanced the total sugar contents in various cell wall fractions. Sugar contents were higher in hemicellulose than in pectin and cellulose. Sugar contents in pectin increased 1.22-fold with 25 µM Cu. The major components of cell wall fractions are galacturonic acids. The content of galacturonic acids was considerably higher than that in the control during Cu treatment, particularly at higher Cu supply. The galacturonic acid values rapidly increased in pectin (1.28-fold), hemicellulose (1.22-fold), and cellulose (1.79-fold) at 100 µM Cu. A conserved disaccharide residue known as 2-keto-3-deoxyoctonic acid (KDO) is attached to C-3 of the backbone of rhamnogalacturonan II in pectin. The KDO concentration in pectin increased during Cu exposure. Thus, the highest concentration of KDO (1.49-fold) was found in the 100 µM Cu-treated group.

**Figure 4:**
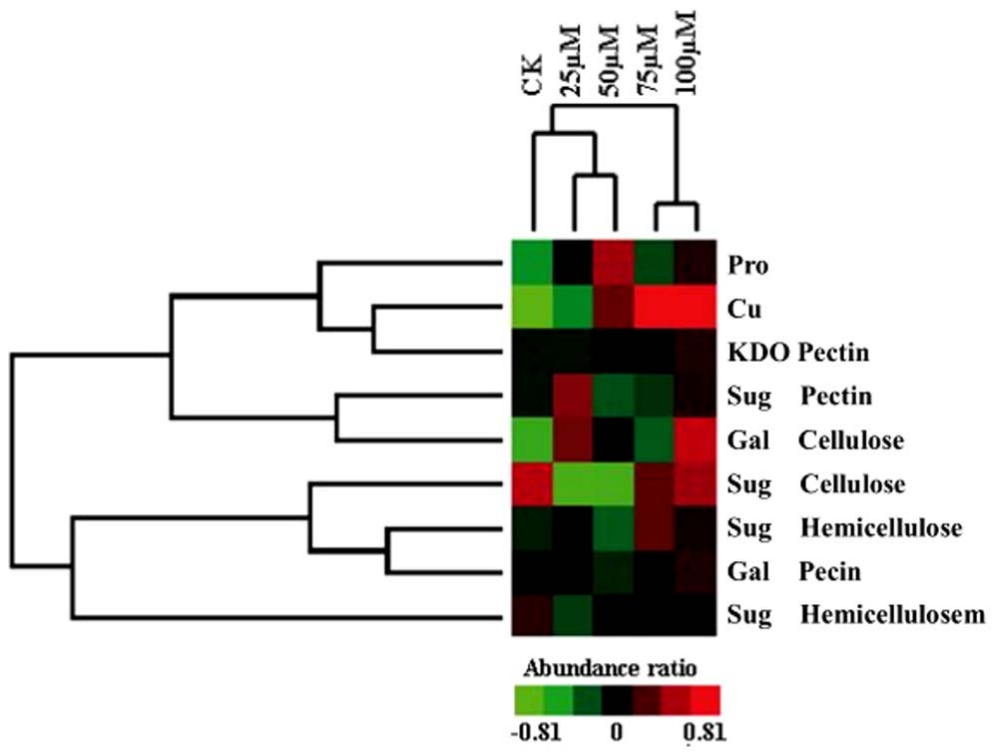
Hierarchical cluster result of cell wall polysaccharide abundance ratio using the average linkage distance between clusters is shown. The color weighting represents normalized levels of each variable from the high (red) to the low (green).

The results indicated that excess Cu affected the intracellular distribution of polysaccharides in the root cell wall. Given this reason, we focused on the relationship between wall polysaccharide composition and CWPs with increasing Cu intensity. The dendrogram proved the connection between the contents of polysaccharides and proteins as matched with different Cu concentrations (Figure 4). This finding suggests that Cu induced changes in the contents of root cell wall polysaccharides and proteins, which altered the intracellular distribution patterns in assigning effects on cellular activities.

### Cu-responsive CWP identification using TMT LC-MS/MS

This study aimed to gain a better understanding of the metabolic processes and molecular mechanisms involved in Cu-responsive CWPs. A TMT-based shotgun quantitation approach was used to obtain an overall view of the proteome changes associated with Cu stress in the root cell wall of *E. splendens*. Unique proteins were successfully identified based on homology searching with a common protein confidence cutoff of 95% and relative quantitative information in control and Cu-treated samples, as indicated by the peak areas of the different TMT tags (Table 1, Table S2). A threshold of 1.5-fold change and a *p*<0.05 were set as stringent criteria for significant differences between control and treatment. Fifty five proteins were classified as differentially expressed upon Cu stress using the criteria; 22 and 33 of these proteins showed increasing trends and decreasing abundance, respectively (Table 1, Table 2).

**Table 1:** Root cell wall proteins with significant increased in expression level under 50 µM Cu treatment identified by LC-ESI-MS/MS-based proteomics using SIEVE (p<0.05 and fold chang >1.5).

Serial No.	Protein name	gi number^a^	Percent coverage b (%)	Fold Change^c^	*p v*alue^d^	Species identified^e^	MW(kDa)	p*I*
1	Chloroplast NAD-MDH	3256066	8.68	1.50	0.00000	Arabidopsis thaliana	49.9	8.2
2	D-glyceraldehyde-3-phosphate Dehydrogenase(GAPDH)	51849658	17.26	1.52	0.00000	Periploca sepium	25.3	6.2
3	40S ribosomal protein S19	74231038	9.32	1.52	0.00271	Solanum peruvianum	15.7	5.5
4	Phosphoglycerate kinase	21272	5.08	1.53	0.00963	Spinacia oleracea	55.1	6.1
5	Putative malate dehydrogenase	50080249	15.29	1.55	0.00346	Oryza sativa Japonica Group	41.6	5.0
6	Cyc07	1856971	6.12	1.56	0.02389	Catharanthus roseus	35.6	6.0
7	2,3-bisphosphoglycerate-independent phosphoglycerate mutase	168588	1.61	1.57	0.00000	Zea mays	68.8	5.2
8	Non-symbiotic hemoglobin	11095158	13.75	1.58	0.01599	Medicago sativa	23.0	9.0
9	P0 ribosomal protein	1143507	6.83	1.59	0.00000	Lupinus luteus	41.0	9.8
10	Putative 40S ribosomal protein s12	643074	7.75	1.60	0.03853	Fragaria x ananassa	21.2	4.7
11	Unknown	255648032	11.18	1.62	0.02313	Glycine max	21.3	6.4
12	Mitochondrial chaperonin	415733	7.16	1.68	0.01830	Brassica napus	74.5	5.4
13	Maturase K	33332509	4.13	1.69	0.01270	Pitcairnia xanthocalyx	48.8	6.9
14	Pectin methylesterase	1222552	2.23	1.70	0.00633	Solanum lycopersicum	75.5	9.5
15	Porin-like protein	47847590	3.61	1.71	0.01348	Oryza sativa Japonica Group	36.1	6.4
16	Pollen allergen Que a 1 isoform	167472851	7.50	1.72	0.00062	Quercus alba	21.3	8.8
17	Putative 1,4-benzoquinone reductase	53749369	5.37	1. 73	0.00000	Oryza sativa Japonica Group	49.9	8.2
18	Thioredoxin h	13624884	9.32	1.89	0.02335	Pisum sativum	25.3	6.2
19	Predicted protein	162680354	5.16	1.91	0.00523	Physcomitrella patens subsp	24.8	8.9
20	Hypothetical protein OsI_31140	125563499	2.51	1.99	0.00035	Oryza sativa Indica Group	16.1	4.7
21	Hypothetical protein OsI_07053	218190658	1.47	2.70	0.00000	Oryza sativ a Indica Group	37.3	5.8
22	60s acidic ribosomal protein P2	551267	7.89	3.31	0.00000	Parthenium argentatum	56.7	5.4

a gi no., gene identification number as in GenBankTM.

b Coverage., sequence coverage.

c Fold change.

d p value, indicates the significance of up- or down- regulation of spots according to the F-test through analysis of variance (p<0.05).

e Species identified by Mascot search using NCBI.

**Table 2:** Root cell wall proteins with significant decreased in expression level under 50 µM Cu treatment identified by LC-ESI-MS/MS-based proteomics using SIEVE (*p*<0.05 and fold chang >1.5).

Serial No.	Protein name	gi number^a^	Percent coverage b (%)	Fold Change^c^	*p v*alue^d^	Species identified^e^	MW(kDa)	p*I*
23	Predicted protein	162671613	1.87	−2.68	0.00000	Physcomitrella patens subsp	71.6	4.9
24	Peroxidase	17066703	2.71	−2.59	0.00000	Capsicum annuum	39.5	6.9
25	Os07g0223100	113610742	18.75	−2.28	0.03704	Oryza sativa Japonica Group	6.1	6.0
26	Mitochondrial phosphate translocator	1842188	2.47	−2.25	0.00466	Betula pendula	45.3	9.4
27	Predicted protein	549049082	3.53	−2.23	0.01662	Populus trichocarpa	30.5	8.9
28	Actin	288300146	16.80	−2.17	0.00166	Cleistogenes songorica	46.2	5.5
29	Ubiquitin fusion protein	149391425	36.04	−2.08	0.02095	Oryza sativa Indica Group	16.6	10.0
30	Heat shock protein Hsp70	124360342	2.59	−1.98	0.02106	Medicago truncatula	80.9	6.4
31	Putative nascent polypeptide associated complex alpha chain	20160782	6.93	−1.97	0.00728	Oryza sativa Japonica Group	26.9	4.4
32	Hypothetical protein SORBIDRAFT_09g020360	241945088	8.50	−1.88	0.00003	Sorghum bicolor	49.1	5.2
33	Plasma membrane-type calcium ATPase	3335060	1.08	−1.86	0.03138	Arabidopsis thaliana	125.3	8.9
34	Cytochome b5	2695711	11.94	−1.83	0.01678	Olea europaea	18.3	5.5
35	Unknown	118482646	3.48	−1.82	0.00000	Populus trichocarpa	41.8	4.9
36	ATP synthase beta subunit	8452631	4.35	−1.80	0.04389	Cichorium intybus	18.1	6.2
37	Peptidyl-prolyl cis-trans isomerase	118104	7.56	−1.76	0.00000	Zea mays L	21.8	9.0
38	Ferredoxin–NADP+ reductase-like protein	7267299	8.61	−1.73	0.00081	Arabidopsis thaliana	48.3	8.9
39	Putative protein	5262759	0.82	−1.71	0.04231	Arabidopsis thaliana	124.3	8.5
40	Conserved hypothetical protein	223517971	3.65	−1.71	0.00000	Ricinus communis	24.2	4.2
41	Unnamed protein product	219742878	7.50	−1.70	0.01691	Glycine max	38.2	5.6
42	Unknown	255634488	7.50	−1.69	0.01879	Glycine max	38.0	5.4
43	Ferredoxin-NADP Reductase	6634773	7.59	−1.69	0.00157	Oryza sativa	50.9	9.2
44	Predicted protein	222844395	1.20	−1.68	0.00000	Populus trichocarpa	72.6	9.2
45	60S ribosomal protein L25	310935	14.94	−1.66	0.00002	Nicotiana tabacum	24.8	8.8
46	Vacuolar H(+)-ATPase subunit-like protein	7801655	2.27	−1.64	0.00000	Arabidopsis thaliana	58.0	5.9
47	Small G protein	974780	4.06	−1.61	0.01530	Beta vulgaris subsp. vulgaris	26.3	10.1
48	Elongation factor 1 subunit alpha	162036292	8.22	−1.58	0.02150	Cenchrus americanus	30.2	6.6
49	2-dehydro-3-deoxy phosphor heptonate aldolase	2546988	5.61	−1.52	0.01476	Morinda citrifolia	65.5	9.3
50	RAS-related GTP-binding protein	20756	5.34	−1.52	0.00000	Pisum sativum	26.7	8.3
51	Aspartic proteinase nepenthesin-1 precursor, putative	223535787	3.05	−1.52	0.00000	Ricinus communis	46.6	6.5
52	Hypothetical protein	147774267	0.73	−1.52	0.00000	Vitis vinifera	24.1	4.9
53	Hypothetical protein VITISV_025412	147822728	8.42	−1.52	0.00000	Vitis vinifera	21.9	7.3
54	Predicted protein	222855760	6.33	−1.51	0.00185	Populus trichocarpa	41.8	5.0
55	Hypothetical protein VITISV_023718	147801802	1.87	−1.51	0.00000	Vitis vinifera	71.1	7.8

a gi no., gene identification number as in GenBankTM.

b Coverage., sequence coverage.

c Fold change.

d p value, indicates the significance of up- or down- regulation of spots according to the F-test through analysis of variance (p<0.05).

e Species identified by Mascot search using NCBI.

### Functional classification of identified CWPs

**Figure 5:**
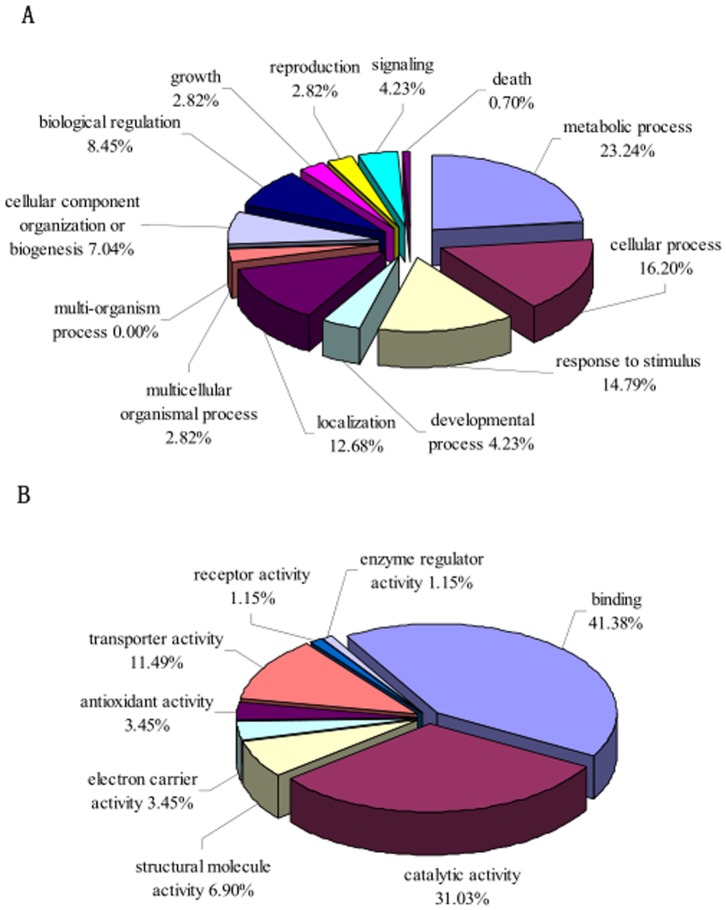
Functional cataloguing of 55 differentially expressed cell wall proteins in *Elsholtzia splendens*'s root based on GO annotation. The pie charts show the distribution of 55 differentially expressed cell wall proteins on of the Cu –responsive proteins into their functional classes in percentage. A: Biological Process Ontology, B: Molecular Function Ontology.

Database search using Uni-Prot/NCBI accession numbers was conducted to obtain detailed information of differentially expressed proteins. These proteins were categorized into biological process and molecular function using an in-house Perl script according to the extracted GO terms from InterPro (www.ebi.ac.uk/interpro/) or Pfam (pfam.sanger.ac.uk/) (Figure 5). Functional classifications of the 55 differentially expressed proteins (Table 1, Table 2) were classified based on the GO terms that correspond to the biological process. The top three categories were those involved in metabolic processes (23.24%), cellular processes (16.20%), and response to stimuli (14.79%) (Figure 5A). These three categories were classified according to the GO molecular annotation into binding (41.38%), catalytic activity (31.03%), and transport activity (11.49%) (Figure 5B). The observed diversity in biological function and processes of the differentially expressed CWPs demonstrated that the response of the root cell wall of *E. splendens* to Cu stress was a complex process. Many physiological and biochemical changes were altered to counteract the adverse conditions.

Specific enzymes constituted by some small differentially expressed CWPs participated in various metabolic processes to counteract Cu stress. Classification pathways according to the KEGG database searches categorized the cell wall enzymes into 12 classes (Table 3, Table S2). The more important category pathways were involved in oxidative phosphorylation, phenylalanine biosynthesis and metabolism, ascorbate and aldarate metabolism, and citrate cycle. All these pathways suggested the relatively altered abundance of the enzymes in various metabolic pathways that probably re-optimized the metabolic processes to combat against Cu stress.

**Table 3:** Category of differentially expressed cell wall proteins refers to the entry on the classification of pathways from KEGG database.

No.	Pathway	Enzyme	Enzyme ID	Change Folds	Protein Name
1	Phenylpropanoid biosynthesis??? phenylalanine metabolism	Lactoperoxidase	ec:1.11.1.7	−2.59	Peroxidase
				−1.69	Ferredoxin-NADP Reductase
				−1.66	Predicted protein
2	Phenylalanine, tyrosine and tryptophan biosynthesis	Synthase	ec:2.5.1.54	−1.52	Hypothetical protein VITISV_025412
				2.70	Hypothetical protein OsI_07053
3	Nitrogen metabolism	(6S)-tetrahydrofolate aminomethyltransferase dehydratas	ec:2.1.2.10	1.72	Pollen allergen Que a 1 isoform
			ec:4.2.1.1	−1.83	Cytochome b5
4	One carbon pool by folate	(6S)-tetrahydrofolate aminomethyltransferase	ec:2.1.2.10	1.72	Pollen allergen Que a 1 isoform
5	Starch and sucrose metabolism	Synthase	ec:2.4.1.34	1.50	Chloroplast NAD-MDH
6	Oxidative phosphorylation	ATPase	ec:3.6.3.6	−1.76	Peptidyl-prolyl cis-trans isomerase
		Dehydrogenase	ec:1.3.5.1	1.53	Phosphoglycerate kinase
7	Ascorbate and aldarate metabolism	Oxidase	ec:1.1.3.8	−1.64	Vacuolar H(+)-ATPase subunit-like protein
8	Glyoxylate and dicarboxylate metabolism	(Si)-synthase	ec:2.3.3.1	−1.86	Plasma membrane-type calcium ATPase
9	Sphingolipid metabolism	Phosphodiesterase	ec:3.1.4.12	−1.52	2-dehydro-3-deoxyphosphoheptonate aldolase
10	Citrate cycle (TCA cycle)	(Si)-synthase	ec:2.3.3.1	−1.86	Plasma membrane-type calcium ATPase
		dehydratas	ec:1.3.5.1	1.53	Phosphoglycerate kinase
11	Glycine, serine and threonine metabolism	(6S)-tetrahydrofolate aminomethyltransferase	ec:2.1.2.10	1.72	Pollen allergen Que a 1 isoform
12	Methane metbolisma	Lactoperoxidase	ec:1.11.1.7	−2.59	Peroxidase
				−1.69	Ferredoxin-NADP Reductase
				−1.66	Predicted protein

## Discussion

### Cell wall polysaccharide remodeling in root under Cu stress

Cell walls provide a physical barrier to plants grown in Cu-contaminated soil, and cell wall polysaccharides have a crucial role in metal binding and accumulation [7]. The binding ability depends on the number of functional groups in polysaccharides, particularly for abundant carboxyl groups [7]. In the present study, the contents of the various cell wall polysaccharides significantly increased with the increase in Cu concentration. The expression of cell wall polysaccharides, which contain more carboxyl groups, provided more binding sites for Cu ions. Excess Cu also affected the intracellular distribution of polysaccharide in the root cell walls (Figure 3). Similar situations were also described in rice roots under Cd stress [45] and *Arabidopsis* roots under Al stress [46]. The starch and sucrose metabolism-related proteins (Table 3) – Chloroplast NAD-MDH (No. 1, Table 1) – were also markedly up-regulated in response to Cu stress based on our observations. These findings suggest that Cu or Cu-induced oxidative stress may activate some crucial enzymes in the glyconeogenesis pathway, which can alter the contents of cell wall polysaccharides.

Increasing physiological, biochemical, and molecular evidence also showed that the remodeling of the binding properties of root apoplasts was attributed to Cu resistance. Enhancement at the level of low-methylesterified pectins, where the polysaccharides bound more trivalent metal ions, is one of the most remarkable alterations. Pectin methylesterases (PMEs), known as pectinesterases, catalyzed the demethylesterification of the homogalacturonan pectin domain in the cell wall. Demethylesterification of the pectin increases the abundance of free carboxylic acid groups on the galacturonic acid residue [47]. A flax PME has been recently implicated in wall remodeling following Cd treatment [48]. Interestingly, this study identified PMEs (No. 14, Table 1) as Cu-induced differentially expressed proteins; the protein had changes in abundance of 1.7-fold (*p*≤0.5). Catalyzed ROS by excess Cu^2+^ can also damage or cause degradation of essential complex molecules in cell wall polysaccharide. Generating hydroxyl radicals from H_2_O_2_ possesses a direct role in cell wall loosening through polysaccharide cleavage [49], [50]. Hydroxyl radicals (˙OH) may cause non-enzymic scission of polysaccharides in vivo (e.g., in plant cell walls) [50]. Basing on these findings, we concluded that the chemical composition and distribution of plant cell wall polysaccharides are a factor in the outcome of plant–metal interaction, which elucidated a possible role in a novel Cu-resistance mechanism.

### Cu-induced oxidative stress and antioxidant defense in cell wall

Cu is a redox active metal that catalyzes ROS production, such as superoxide (O_2_ ˙), hydrogen peroxide (H_2_O_2_), and hydroxyl radicals (OH·) through Habere Weiss and Fenton reactions [51]. ROS can act as signaling molecules for stress response. However, ROS can cause damage to many cellular components above a certain threshold. Most Cu-tolerance mechanisms are primarily involved in protecting the cellular structure. An important method is the control of the level of ROS or the limitation of damage caused by ROS. In the current study, significant changes in the abundance of some antioxidant and defense-related proteins suggested that ROS can be involved in a Cu-induced oxidative stress response. Analysis of *E. splendens* root CWPs confirmed the associated gene expression for enzymes involved in ROS scavenging and oxidative phosphorylation (Table 3), such as peroxidase (No. 24, Table 2), peptidyl-prolyl cis-trans isomerase (No. 37, Table 2), and phosphoglycerate kinase (No. 4, Table 1). Among these proteins, peptidyl-prolyl cis-trans isomerase, as a protein chaperone, possess complementary and sometimes overlapping roles in protecting the proteins [15].

Excess Cu generates oxidative stress, thereby hindering some important metabolic process, such as up-regulation of antioxidant and stress-related regulatory proteins, that help maintain cellular homeostasis [52]. Maturase K (No. 13, Table 1) changed in abundance of 1.69-fold. Maturase K has an important role in plant growth, cell division, and expansion as well as in protecting metabolic processes against H_2_O_2_ and other toxic derivatives of oxygen. Hydroquinone formation was accompanied by the oxidation of two moles of NADPH and the presence of an inducible 1,4-benzoquinone reductase. Thus, putative 1,4-benzoquinone reductase (No. 17, Table 1) plays an essential role in antioxidant defense pathway [53]. Studies also reported about wall-bound malate dehydrogenase (No. 5, Table 1) [54], [55], which can regenerate NAD(P)H that is needed by cell wall peroxidases for free radical generation associated with lignin polymerization. Thioredoxin (No. 18, Table 1) is also involved in redox regulation by reducing disulfides on the target protein for detoxifying lipid hydroperoxides or repairing oxidized proteins and relaying the signal to mitogen-activated protein kinase pathway of stress signaling [56]. Phosphoglycerate kinase (No. 4, Table 1) can also interact with cytosolic catalase and has a role in relieving oxidative stress. Interestingly, down-regulation of key metabolic enzymes revealed that oxidant protection conferred by these proteins was also regulated during Cu treatment. Ferredoxin-NADP reductase (No. 43, Table 2) and ferredoxin-NADP reductase-like protein (No. 38, Table 2), known to sequester highly reactive Fe^3+^ and prevent formation of toxic ˙ OH species, were also identified [57]. Vacuolar H(+)-ATPase subunit-like protein (No. 46, Table 2) was involved in ascorbate and aldarate metabolism. L-ascorbic acid is characterized by plant tissues, and ascorbate is one of the most important antioxidant molecules [58]. Furthermore, 2-dehydro-3-deoxy-phosphorheptonate aldolase synthase (No. 49, Table 2) may be involved in aromatic pathways in the secondary metabolites, which are known to act as defense responses to abiotic stress [59].

### Cu stress-activated signaling pathways

The communication between the cytoskeleton and the CWPs is one of the most characterized features of cellular mechanisms that enable cells to respond effectively to various extracellular signals. Several candidate components involved in signal transduction were identified in this study, including Hsp70, small G protein, and cytochrome. The biosynthesis and accumulation of HSP proteins (No. 30, Table 2) can generally contribute to the protection and repair of cells under stress [60]. These proteins may be involved in cell wall biogenesis [61]. Small G proteins (No. 47, Table 2) transduced signals from receptors to control a wide range of cellular functions, particularly for regulating Ca^2+^ channel expression at the cell surface [62]. These proteins are clustered into distinct families but all act as molecular switches, which are active in their GTP-bound form but inactive when GDP-bound [63]. RAS-related GTP-binding protein (No. 50, Table 2) also has a primarily role in modulating cellular functions that involve actin cytoskeleton (No. 28, Table 2), such as establishing cell polarity and morphology [64]. Cytochrome has been identified in the stem cell wall of *Medicago sativa* [65] and germinating embryos of *Oryza sativa* [66]. This first proteomic study showed that cytochromes (No. 6, Table 1; No. 34, Table 2) were Cu-responsive proteins in the root cell wall of *E. splendens*. More preliminary evidence suggested that porins can form aqueous transmembrane channels for transporting solutes and macromolecules across the extracellular surface [67] [68]. These pores are regulated by ATP and GTP with a gating mechanism that modulates the pore size and ion selectivity [54]. In this case, Cu resistance mechanism may be induced by active apoplastic permeability because of up-regulated porin-like proteins (No. 15, Table 1).

### Cu stress-activated energy pathways and protein synthesis

ATP is an essential metabolite in cell walls associated with energy conversion [69]. The plasma membrane calcium ATPase (PMCA) (No. 33, Table 2) or Ca^2+^ pump transports Ca^2+^ ions out of the cells using the stored energy in ATP. Control of Ca^2+^ concentration is significant in the cytosol [70]. PMCA down-regulation at the protein levels was observed during the experiment, which may represent an adaptive mechanism to facilitate removal of Ca^2+^ in the maintenance of calcium homeostasis in abiotic stress. The vacuolar H(+)-ATPase (No. 46, Table 2) functions as a primary proton pump that generates electrochemical gradients of protons across the transmembrane region [71], which provides the primary driving force for transporting numerous ions and metabolites against their electrochemical gradients [72]. Glyceraldehyde-3-phosphate dehydrogenase (GAPDH) (No. 2, Table 1), as a classical cellular enzyme involved in glycolysis, is also differentially expressed in response to Cu exposure, where GAPDH is specifically targeted at the cell wall [73]. Mitochondrial phosphate translocator (No. 26, Table 2), involved in ATP/ADP transportation, is also identified in the cell wall. Amino acids and amino acid-derived molecules can be chelated with metal ions by high affinity ligands [74]. Pollen allergen Que a 1 isoform (No. 16, Table 1), which is involved in glycine, serine, and threonine metabolism (Table 3), was up-regulated in the root cell wall of *E. splendens*, whereas aspartic proteinase nepenthesin-1 precursor (No. 51, Table 2) was down-regulated by Cu stress. Different expression levels of amino acid-related proteins in the cell wall elucidated their possible role in Cu tolerance.

### Other Cu stress-responsive proteins and their potential functions

The 2,3-bisphosphoglycerate independent phosphoglycerate mutase (No. 7, Table 1) was up-regulated in the study, which was also differentially expressed in rice anthers under cold stress [75]. Previous evidence indicated that the expression of non-symbiotic hemoglobins (nsHbs) from cotton is induced with Verticillium wilt fungus [76]. The proteins also had a role in the defense responses against pathogen invasions in *Arabidopsis* [76]. Our studies showed that the nsHbs (No. 8, Table 1) were up-regulated in the cell wall of *E. splendens* root under Cu stress, although their physiological function is yet to be determined. A significant amount of the unidentified proteins were classified as hypothetical or predictable. These proteins were annotated in databases as unknown, hypothetical, or putative proteins because of the theoretical translation of open reading frame sequences [77]. The roles of these proteins remain to be investigated.

### Overview of regulated CWP functions

**Figure 6:**
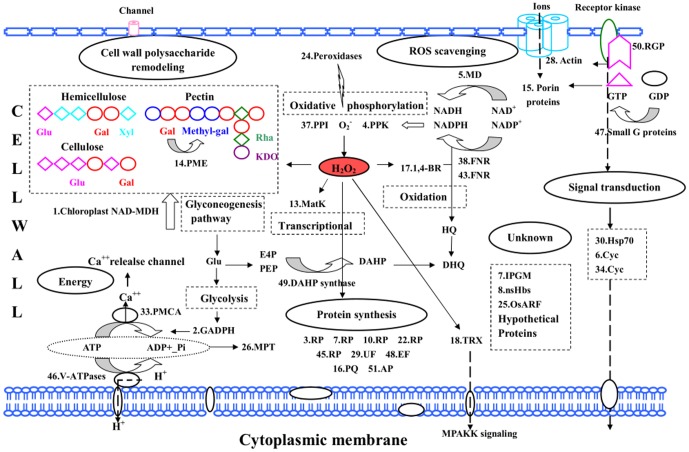
Pathways involved in cell defense, signaling, and cell wall remodeling under copper stress in the root cell wall of *Elsholtzia splendens*. Proteins identified in this study are displayed on the corresponding metabolic pathways and the number indicates the protein identification number. Gal, galacturonic acids; Methyl-gal, methylated galacturonic acids; KDO, 2-keto-3-deoxyoctonic acid; Rha, rhamnose; PME, Pectin methylesterase; Xyl, xylanase; Glu, glucose; MD, malate dehydrogenase; Trx, thioredoxin; MAPKK, mitogen-activated protein kinase kinase; PPI, peptidyl-prolyl cis-trans isomerase; PPK, phosphoglycerate kinase; MatK, maturase K; 1,4-BR, putative 1,4-benzoquinone reductase; oxidation; FNR, ferredoxin-NADP reductase; HQ, hydroquinone; DHQ, Durohydroquinone; DAHP, 3-deoxy-D-arabino-heptulosonic acid-7-phosphate; E4P, perythrose-4-phosphate; PEP, phosphoenolpyruvate; RGP, RAS-related GTP-binding protein; PLP, porin-like proteins; Cyc, cytochrome; PMCA, plasma membrane calcium ATPase; V-ATPases, vacuolar H(+)-ATPases; MPT, mitochondrial phosphate translocator; GADPH glyceraldehyde-3-phosphate dehydrogenase; RP, ribosomal protein; UF, ubiquitin fusion protein; EF, elongation factor 1 subunit alpha; PQ, Pollen allergen Que a 1 isoform; AP, aspartic proteinase nepenthesin-1 precursor; nsHbs. non-symbiotic hemoglobin; OsARF, Os070223100; iPGM, 2,3-bisphosphoglycerate-independent phosphoglycerate mutase.

This study provides insights into the functional role of the cell wall of *E. splendens* under Cu stress, where several CWPs were identified with possible roles in Cu tolerance/detoxification. Cu stress conditions can alter the composition (polysaccharide and protein) of the cell wall both qualitatively and quantitatively. Approximately 40% of the differentially expressed CWPs showed higher abundance in response to Cu stress involved in antioxidant defense, cell wall polysaccharide remodeling, and metabolism process. Up to 60% of the CWPs were in low abundance in response to Cu stress that is involved in signal, energy, and protein synthesis. Polysaccharide analysis confirmed the cell wall remodeling under Cu stress. The amount, composition, and distribution of the cell wall polysaccharides are consequential for plant adaptation to enhance Cu ion levels. Proteome analysis of cell wall confirmed that most proteins were associated with an antioxidant defense response. Hsp 70, small G protein, and RAS-related GTP-binding protein also have essential roles in signal transduction across the cell wall and through the cytoskeleton. Literature provides fundamental information about the role of polysaccharide composition of plant cell wall in metal tolerance and complementary evidence on continuous crosstalk between CWPs and the cytoskeleton. Therefore, knowledge has been expanded on plant stress-related signaling pathways in the cell wall. Cu regulation of these proteins may also not solely respond on abundance changes. Post-translational modification and dynamics are interesting subjects for future investigation. The present study is the first cell wall proteome and polysaccharide investigation of plants in response to Cu and important for the understanding of the plant cell wall response to environmental heavy metal stresses. We propose a possible protein interaction network (Figure 6) that provides new insights into Cu stress response in the root cell wall and facilitates further functional research of target proteins that are associated with Cu response based on the abundant changes in these proteins, as well as polysaccharides and their putative functions.

## Materials and Methods

### Ethics statement

No specific permissions were required for collecting *E. splendens* seeds from deposited Cu-mining soil in Zhuji County, Zhejiang Province, China. *E. splendens* is neither endangered nor protected. Authors maintained the population at sustainable levels. All plant work was conducted according to relevant national and international guidelines.

### Plant material and Cu treatment

*E. splendens* seeds were collected from plants that grew on deposited Cu-mining soil in Zhuji County, Zhejiang Province, China. The seeds were washed with deionized water and soaked in distilled water to germinate in a controlled dark condition (25°C) for 48 h. The solution was changed to one-quarter-strength complete nutrient solution after germination. At the fourth leaf stage, uniform seedlings were transferred into vessels filled with full-strength nutrient solution that contained macronutrients (in mM): 1.0 Ca(NO_3_)_2_, 0.5 MgSO_4_, and 0.5 K_2_HPO_4_; and micronutrients (in µmol/L): 27.0 Fe(III)–EDTA, 23.0 H_3_BO_3_, 0.8 CuSO_4_, 0.5 Na_2_MoO_4_, 0.5 ZnSO_4_, and 4.5 MnSO_4_. The pH was adjusted to 5.8 with 0.1 M HCl and NaOH. Nutrient solutions were renewed every 3 d and aerated continuously through 0.2 µm filters. Plants were grown in a hydroponic nutrient solution chamber with a 16 h, 25°C day and an 8 h, 20°C night regimen at 60% to 70% relative humidity. Light conditions in the growth chamber were fixed at 5 µmol to 10 µmol photons m^−2^ s^−1^. Plants were exposed to various concentrations of Cu (25, 50, 75, and 100 µM) after 28 d of growth, which was added as sulfate for 48 h. Each treatment (15 plants) was conducted in triplicate, and the control plants (CK) were grown in pure nutrient solution for comparison. Plant roots were rinsed with distilled water at harvest and then immersed in 5 mmol/L of Ca(NO_3_)_2_ for 20 min to remove the putative adsorbed Cu^2+^ [32]. Roots were separated, pooled, and rinsed with deionized water; plant parts were washed, immediately frozen in liquid nitrogen, and stored at −80°C for analysis [33].

### Cell wall preparation

Cell wall purification and CWP extraction were prepared as described by Feiz et al. [34] with slight modifications. All procedures were conducted at 4°C unless mentioned otherwise. Fifteen independent root preparations were pooled to yield one biological replicate. Root tissues (4 g) were homogenized in pre-cold extraction buffer [5 mM acetate, 0.4 M sucrose, 1 mM phenylmethylsulfonyl fluoride (PMSF), pH 4.6, 125 mL] with a chilled mortar and pestle. After adding 0.4 g of polyvinylpolypyrrolidone (PVPP), the mixture was incubated at 4°C for 30 min with stirring. Cell walls were separated from soluble cytoplasmic fluid by centrifuging the homogenate at 1,000×*g* for 15 min. The pellet was washed by suspension in 125 mL of 5 mM acetate buffer at pH 4.6 with 0.6 M sucrose. The mixture was centrifuged at 1,000×*g* for 15 min. The pellet was further purified by resuspension in 125 mL of 5 mM acetate buffer at pH 4.6 with 1 M sucrose and centrifuged at 1,000×*g* for 15 min. The residue was washed thoroughly with 750 mL of 5 mM acetate buffer at pH 4.6 while filtered on a layer of Miracloth (Merck, Darmstadt, Germany). The supernatant was discarded, and the final pellet was freeze-dried overnight. The freeze-dried cell wall materials were then stored at −80°C for further use. Most intracellular proteins were removed from the cell walls given the advantage of sucrose gradients and extensive washing with low ionic strength acidic buffer. PVPP was treated with acid to increase polymerization and to remove metal ions and contaminants.

### Cell wall composition extraction and analysis

#### Isolation of cell wall material

Cell wall materials were extracted according to Zhong and Lauchli (1993) [35]. The pectin fraction was extracted twice with 0.5% ammonium oxalate buffer that contained 0.1% NaBH_4_ (pH 4) in boiling water bath for 1 h each and pooled the supernatants. Pellets were subsequently subjected to triple extractions with 4% KOH that contained 0.1% NaBH_4_ at room temperature for 24 h, followed by similar extraction with 24% KOH that contained 0.1% NaBH_4_. The supernatants from the 4% and 24% KOH extractions were collected and thus yielded the hemicellulose fractions. The remaining pellet from the 24% KOH extraction was then lyophilized, weighed, and considered to be the cellulose fraction [36].

#### Protein extraction and digestion

A portion (2 g) of the roots was homogenized with 1 mL of PBS (pH 7.6) that contained 65 mM K_2_HPO_4_, 2.6 mM KH_2_PO_4_, 400 mM NaCl, and 3 mM NaN_3_ in a mortar and pestle. The homogenate was centrifuged twice at 15,000×*g* for 10 min, and the supernatant was collected as the total soluble protein. CWPs were extracted from the root cell wall fraction in two successive steps. The first step used CaCl_2_ solution (5 mM sodium acetate buffer, pH 4.6, 0.2 M CaCl_2_, and 1 mM PMSF; CaCl_2_ can efficiently extract CWPs that exert weak electrostatic interactions with other cell wall components [37]). The second step involved two extractions with NaCl solution (5 mM sodium acetate buffer, pH 4.6, 1 M NaCl, and 1 mM PMSF; NaCl was also used to extract the strong ionically bound proteins) [34]. For each extraction, the sample was incubated with vortexing at 4°C, and the supernatant was collected after centrifugation at 4,000×*g* for 15 min. The supernatants from all the extraction steps were pooled and concentrated to 4 mL by centrifugation at 1,500×*g* at 10°C using a 3 kDa molecular weight cut-off Amicon Spin Tube (Millipore, MA). The protein sample was buffer-exchanged with ultrapure water.

CWP extracts were dissolved in 100 µL of lysis buffer (7 M urea, 2 M thiourea, 5 mM EDTA, 10 mM DTT, and 1 mM PMSF). The supernatant was transferred to a new tube, reduced with 10 mM DTT for 1 h at 56°C, and alkylated with 55 mM iodoacetamide for 45 min at room temperature in darkness. The protein was precipitated with four volumes of pre-chilled acetone for 30 min at −20°C. The pellet was dissolved in 0.5 M TEAB after centrifugation and sonicated for 5 min. The centrifugation step was repeated, and the supernatant was collected. Approximately 100 µg of proteins from each sample was digested with trypsin (Promega) overnight at 37°C in a 1∶20 trypsin-to-protein mass ratio.

#### Analysis of Cu, protein, and cell wall polysaccharide content

Cell wall fraction was dried at 70°C to a constant weight and then digested at 145°C for 24 h in an acid mixture of HNO_3_:HClO_4_ (3∶1, v:v). Cu concentrations were measured by Elmer flame atomic absorption spectrometry (AAS-3600).

Protein contents were determined by the Bradford method [38] with bovine serum albumin as the standard. CWPs were dissolved with 400 µL of SDS sample lysis buffer by boiling for 5 min and then loaded onto a 12% acrylamide mini-gel (5 cm to 8 cm) for 1D SDS-PAGE.

The contents of total sugars, galacturonic acids, and KDO were determined colorimetrically by sulfate-phenol [39], hydroxydiphenyl [40], and thiobarbituric acid [41], respectively.

### Labeling TMT reagents

Peptide was desalted by Strata XC18 SPE column (Phenomenex) and vacuum-dried after trypsin digestion. Peptide was reconstituted in 0.5 M TEAB and processed according to the protocol for 6-plex TMT reagent kits. In brief, two units of TMT reagent (defined as the amount of reagent required to label 100 µg of protein) was thawed and reconstituted in 41 µL of acetonitrile. Peptide samples from 50 µM Cu were treated for 48 h, and CK samples were labeled with TMT tags 126, 128, and 130 as well as TMT tags 127, 129, and 131. Both samples were incubated at room temperature for 2 h. The peptide mixtures were then pooled, desalted, and dried by vacuum centrifugation.

### LC–ESI–MS/MS analysis by Q Exactive

The labeled peptide was resuspended in buffer A (2% ACN, 0.1% FA) and centrifuged at 20,000×*g* for 2 min. The supernatant was transferred into a sample tube and loaded onto an Acclaim PepMap 100 C18 trap column (75 µm×2 cm; Dionex) by EASY nLC1000 nano UPLC (Thermo). The peptide was then eluted onto an Acclaim PepMap RSLC C18 analytical column (50 µm×15 cm; Dionex). An 85 min gradient program was run at 300 nL/min, which started from 3% to 35% B (80% ACN, 0.1% FA), followed by 5 min linear gradient to 90% B, and maintained at 90% B for 5 min.

The peptides were subjected to NSI source, followed by tandem mass spectrometry (MS/MS) in Q Exactive (Thermo) coupled online to the UPLC. Intact peptides were detected in the Orbitrap at a resolution of 70,000. Peptides were selected for MS/MS using 27% NCE with 12% stepped NCE; ion fragments were detected in the Orbitrap at a resolution of 17,500. A data-dependent procedure that alternated between one MS scan followed by 20 MS/MS scans was applied to the top 20 precursor ions above a threshold ion count of 3E4 in the MS survey scan with 5.0 s dynamic exclusion. The applied electrospray voltage was 1.8 kV. Automatic gain control was used to prevent overfilling of the ion trap; 1E5 ions were accumulated for generation of MS/MS spectra. The m/z scan range was 350 Da to 2000 Da for MS scans.

### Proteomic database search

The instrument data file (.raw) was merged and transformed to an.mgf file by Proteome Discoverer (ver. 1.3.0.339; Thermo). Peptide and protein identifications were performed using the Mascot search engine (ver. 2.3.02; Matrix Science). Derived protein sequences from plants in the NCBI were collected, and a database containing 1,596,443 sequences was created. In the Mascot search engine version 2.3.02 software, all parameters were set as follows. Database searching was restricted to tryptic peptides. Carbamidomethyl (C), TMT 6-plex (N-term), and TMT 6-plex (K) were selected as fixed, and deamidated (NQ), Gln→pyro-Glu (N-term Q), and oxidation (M) were selected as variable modifications, where two missed cleavages were allowed with precursor error tolerance at 10 ppm and fragment deviation at 0.02 Da. The Mascot search results were quantified using Mascot 2.3.02 with the following criteria: protein ratio type  =  median, minimum unique peptides  = 1, peptide threshold type  =  at least homolog. Peptides were not quantified if peptide score was too low or the deviation was too large. The final ratios of protein were then normalized by taking the median of all the proteins quantified. The complete list of identified peptides was encoded in an Excel (Microsoft) database for grouping of results into proteins and calculation of ratios and coefficient of variation.

To obtain the differentially expressed proteins, we selected the proteins according to the following principles. (a) The expression change of protein folds is more than 1.5. (b) The protein quantitative T-test *p* value is less than 0.05. A total of 55 differentially expressed proteins were obtained in our bioinformatics analysis data.

### Bioinformatics studies and statistical analysis

Data were the average of at least three independent experimental replicates. One-way ANOVA (Duncan's test) and LSD test with *p*<0.05 as the significance level were performed to analyze the differences between the control and treatments. All data were presented as the mean value ± standard error and were analyzed using SPSS statistical software package (version 16.0).

The clustering of content abundance profiles was performed using Cluster 3.0. Euclidean metric was used for computing the distance between points and cluster centroids, which is a typical choice for K-means clustering analysis. The output was visualized using TreeView after hierarchical clustering.

GO information was used to categorize the biological processes of identified proteins from TMT data sets. GO annotations were extracted from the Uni-Prot database and were matched with corresponding gene locus identifiers embedded in the NCBI RefSeq database. Proteins were then classified based on their biological process using the Web Gene Ontology Annotation Plot [42] (http://wego.genomics.org.cn/cgi-bin/wego/index.pl). Functionally analyzed Cu stress-induced CWPs in biosynthesis pathways were identified through the Kyoto Encyclopedia of Genes and Genomes (KEGG) [43]. These pathways were classified into hierarchical categories according to the KEGG website (http://www.genome.jp/kegg/). Subcellular locations were predicted using TargetP (http://www.cbs.dtu.dk/services/TargetP/), Predotar (http://urgi.versailles.inra.fr/predotar/predotar.html), and WoLF PSORT (http://wolfpsort.org/) (http://www.genscript.com/psort/wolf_psort.html)software applications as previously described [44].

## Supporting Information

Figure S1 **SDS–PAGE of root cell wall proteins under different copper stress.**
(DOC)

Table S1 **Protein subcellular localization and go annotation of 55 significantly differentially expressed proteins.**
(XLS)

Table S2 **Details of 55 significantly differentially expressed proteins identified by TMT using ANOVA including their** ***p*** **–value.**
(XLS)
