# Supplementary figures and images for: New Insights into Regulation of Proteome and Polysaccharide in Cell Wall of Elsholtzia splendens in Response to Copper Stress
Source: PLoS One. 2014 Oct 23;9(10):e109573. doi: 10.1371/journal.pone.0109573 (PMC4207692; doi:10.1371/journal.pone.0109573)

**Figure S1** SDS–PAGE of root cell wall proteins under different copper stress


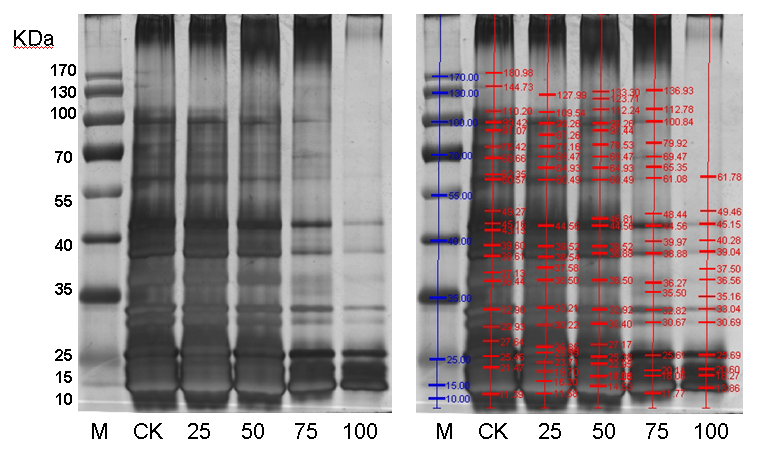

Supplement: Figure S1 — SDS–PAGE of root cell wall proteins under different copper stress. (DOC) [file pone.0109573.s001.doc]
